# Oviposition preference not necessarily predicts offspring performance in the fall armyworm, *Spodoptera frugiperda* (Lepidoptera: Noctuidae) on vegetable crops

**DOI:** 10.1038/s41598-021-95399-4

**Published:** 2021-08-05

**Authors:** Paola Sotelo-Cardona, Wen-Po Chuang, Mei-Ying Lin, Ming-Yao Chiang, Srinivasan Ramasamy

**Affiliations:** 1 World Vegetable Center, 60 Yi-Min Liao, Shanhua, Tainan, 74151 Taiwan, ROC; 2 National Taiwan University, No. 1, Sec. 4, Roosevelt Rd., Taipei, 10617 Taiwan, ROC; 3 Taiwan Agricultural Research Institute, Council of Agriculture, No.189, Zhongzheng Rd., Wufeng Dist., Taichung City, 413008 Taiwan, ROC

**Keywords:** Entomology, Biotic

## Abstract

Given the new spread and potential damage of the fall armyworm (FAW), *Spodoptera frugiperda* (J. E. Smith) (Lepidoptera: Noctuidae) in Asia, it has become imperative to understand the development biology of this invasive species on selected vegetable crops in newer geographical regions. In this study, we investigated the ovipositional preference of FAW females on different host plants, under choice- and non-choice tests. In addition, using the age-stage, two-sex life table theory, we assessed the performance of immature FAW individuals fed and reared on selected vegetable crops to get information related to development time, survival, reproduction and longevity. Fall armyworm females had an oviposition preference on maize compared to other vegetable crops, including cabbage and soybean, and reluctance for tomato, which was confirmed during the choice and non-choice tests. In contrast to the oviposition preference, our results also suggest that despite low preference for cabbage, soybean, and tomato, these crops seemed to provide a high benefit for an appropriate offspring performance, exceeding in some cases the benefits from a maize-based diet. Information from this study was discussed in terms of FAW ecology and how female’s decision affects their reproductive fitness, and the survival and performance of its offspring.

## Introduction

After being initially reported in Africa back in 2016^1^, the fall armyworm (*Spodoptera frugiperda*; FAW) has moved rapidly across many regions including West and sub-Saharan Africa, India, China, Japan, South Korea, Taiwan, and Australia in less than 4 years^2–5^. Besides typical wind-assisted migration (i.e., monsoon winds)^6^, FAW movement across continents has been facilitated by regional transport systems of commodities^7,8^. FAW lacks overwintering capacity and its distribution is mostly limited by cold temperature^9^, with sporadic and seasonal occurrence across tropical and subtropical regions in America occurring via new populations that migrate from overwintering locations in southern Florida and southern Texas–Mexico^10^. In contrast, climate conditions in sub-Saharan Africa, South- and Southeast Asia are highly suitable for establishment of year-round FAW populations (4–6 generations/year)^11,12^.

In terms of economic damage caused by FAW, up to 73% yield losses has been reported in maize in Latin America^13^. In the case of Africa, an early prediction suggested that in the absence of control measures, damage by FAW may cause 21–53% losses in annual maize production in the region, and accounting for more than approximately 21 million tons of maize^14^. In addition, FAO has listed the FAW as a potential food chain threat for several regions in Asia^15^. However, current statistics in Asia are scarce due to the recent spread. So far, China has reported up to 5% of yield losses in the South corn producing region^4^.

Although FAW is a polyphagous pest, infestation occurs primarily on crop grasses from the family Poaceae (Gramineae), including rice, maize, sorghum, wheat, oats, pasture grasses, etc.^16,17^. Other non-gramineous crops that constitute part of FAW diet includes crops in the families Asteraceae (i.e., marigold, pyrethrum, lettuce, sunflower, etc.) Fabaceae (i.e., peanut, chickpea, soybean, etc.), Brassicaceae (i.e., broccoli, cabbage, field mustard, etc.), among others^16^.

Given the new spread and potential damage of the FAW in Asia, it has become imperative to understand the development biology of this invasive species on selected vegetable crops in newer geographical regions. In the particular case of Taiwan, FAW populations were first found in west-central Taiwan in June 2019 in a maize field^18^. In terms of the two strains of FAW in the Americas (i.e., corn and rice strains, based on host preference), the presence of hybrid population has been also confirmed in studies conducted in Africa and Asia. Moreover, a recent paper published by Haineger et al.^19^ confirmed that FAW populations in West Africa (i.e., Benin and Nigeria) consisted of corn-strain individuals and descendants of interstrain hybrids, but not of pure rice-strain individuals, which is also in accordance with previous studies from Nagoshi^20^ where the author suggested the rice strain is either rare, with less than 1% of the population or absent in Africa. More recently, a genome-wide sequencing analysis revealed that FAW populations invading China have a dominant percentage of the corn-strain background and less of rice-strain genetic background^21^.

Based on the polyphagous nature of the FAW, this study aimed to understand (1) female ovipositional preferences and (2) immature survival and development when feeding on alternative plant resources. Therefore, oviposition preference test for FAW female adults as well as life table analysis of different biological traits and demographic parameters of FAW using the age-stage, two-sex life table analysis would allow us to broaden our understanding in terms of adult preference and larval performance in selected vegetable crops.

In terms of ovipositional preference, the preference-performance hypothesis^22,23^ suggest that females will choose to oviposit in hosts with the highest nutritional quality for offspring, and hence, the selected host would allow the offspring to shorten the developmental time, increased biomass, as well as the reproductive potential. However, previous papers also indicate that a potential conflict may arise between parents and offspring, on which larval stage may prefer to feed on a wider range of host plants compared to plants that are actually used as ovipositional substrates for females. These results would suggest that host range would be mostly limited by female behavior and not necessarily due to larval feeding capacities and survival^24–26^. Therefore, information from this study would allow us to have a better understanding on FAW ecology and how female’s decision affect their reproductive fitness, and the survival and performance of its offspring.

## Results

### Oviposition preference: free choice test for fall armyworm females

Fall armyworm female adults significantly preferred maize plants for oviposition compared to other crops under a free choice arena (Table 1). Furthermore, the amount of egg masses (F_3,27_ = 13.27; *P* < 0.0001) and mean number of eggs (F_3,27_ = 17.01; *P* < 0.0001) was significantly higher in maize plants followed by soybean and cabbage. No egg masses as well as eggs from FAW were found in tomato plants when given other vegetables as a choice.

**Table 1:** Average number of egg masses and eggs (mean ± SD, min.–max. range) laid by females of the fall armyworm, *Spodoptera frugiperda* and recorded under a choice test on cabbage, maize, soybean and tomato.

Treatments	Egg masses	Egg numbers
Cabbage	0.57 ± 0.73 (0–2) b	52.57 ± 90.56 (0–266) b
Maize	3.71 ± 1.75 (2–7) a	682.57 ± 341.72 (313–1277) a
Soybean	1.28 ± 1.28 (0–3) b	204.86 ± 249.98 (0–684) b
Tomato	0 b	0 b

Means followed by the same letter(s) in a column are not significantly different (*P* < 0.05) by Tukey’s HSD.

### Fall armyworm oviposition behavior under a no-choice scenario

**Figure 1:**
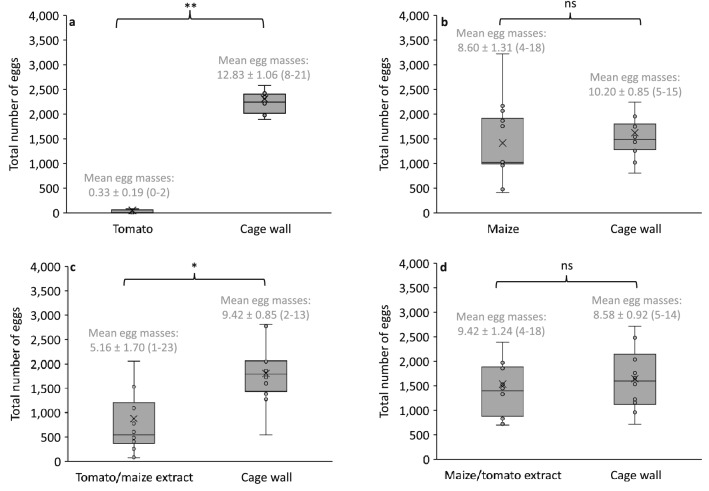
Total number of eggs and egg masses (mean ± SD, min.-max. range) of the fall armyworm, *Spodoptera frugiperda* recorded under a no-choice experiment arena and using (**a**) tomato, (**b**) maize, (**c**) tomato sprayed with maize extract, and (**d**) maize sprayed with tomato extract. Statistical differences based on t-test for the occurrence of egg masses in the plants vs. oviposition observed in the net walls. Statistics for total number of eggs: Tomato (t = 17.02, 11 df, P < 0.001); Maize (t = 0.63, 11 df, P = 0.2697); tomato with maize leaf extract (t = 2.71, 11 df, P = 0.01); Maize with tomato leaf extract (t = 0.31, 11 df, P = 0.378); and for egg masses: Tomato (t = 11.047, 11 df, P < 0.001); maize (t = 0.92, 11 df, P = 0.188); tomato with maize leaf extract (t = 2.052, 11 df, P = 0.0324); maize with tomato leaf extract (t = 0.4597, 11 df, P = 0.3273); **P < *0.001*; *P < 0.05; NS *P* > 0.05.

Under a no-choice scenario, FAW females were reluctant to lay their eggs in tomato, but in contrast significantly more eggs were recorded from the net wall of the cage used for the experiment (Fig. 1a). No differences were observed for the number of eggs laid in maize plants compared to those laid in the cage wall (Fig. 1b). When the “masking” effect was estimated, FAW females still preferred to lay their eggs in the cage wall compared to the tomato sprayed with maize extract (Fig. 1c). In contrast, maize plants sprayed with tomato extract did not deter the preference of females for oviposition (Fig. 1d).

### Life table analysis

**Figure 2:**
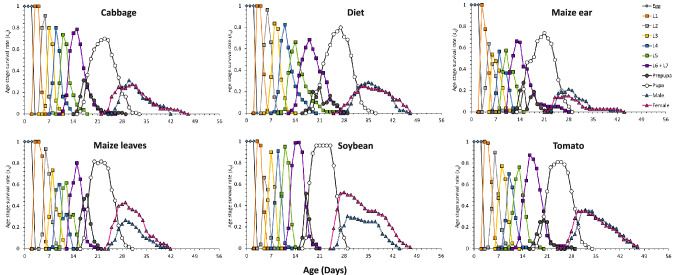
Age-stage-specific survival value (s_*xj*_) of the fall armyworm, *Spodoptera frugiperda* fed on cabbage, maize ear, maize leaves, artificial diet, soybean, and tomato.

The results of development time, survival, reproduction and longevity of FAW fed on cabbage, maize (ear and leaves), soybean, tomato, and artificial diet are represented in Table 2. No statistical analysis was conducted on egg stage, since the study started with new larval hatching at the same time, and 3 days after oviposition. L1 stage was significantly shorter in cabbage, soybean and maize ear treatments, compared to maize leaves. L2 stage was significantly shorter in soybean compared to artificial diet. L3, L4, L5, L6 + L7 stages were significantly shorter in maize (ear and leaves) compared to artificial diet. In addition, the pre-pupal stage was shorter in soybean-reared individuals compared to maize leaves-reared individuals, whereas the pupal stage was significantly shorter in maize leaves compared to artificial diet and maize ear treatments (Table 2, Fig. 2).

**Table 2:** Development time and adult longevity (mean ± SE) of the fall armyworm, *Spodoptera frugiperda* fed on cabbage, maize ear, maize leaves, artificial diet, soybean, and tomato.

Parameter	Cabbage		Maize ear		Maize leaves		Diet		Soybean		Tomato	
	*n*	Days	*n*	Days	*n*	Days	*n*	Days	*n*	Days	*n*	Days
Egg	80	3.00 ± 0.00	80	3.00 ± 0.00	60	3.00 ± 0.00	80	3.00 ± 0.00	80	3.00 ± 0.00	80	3.00 ± 0.00
L1	79	2.19 ± 0.04 d	80	2.15 ± 0.09 d	60	2.93 ± 0.06 a	80	2.38 ± 0.06 c	80	2.30 ± 0.06 cd	80	2.72 ± 0.07 b
L2	76	1.92 ± 0.06 b	80	1.70 ± 0.06 c	59	2.07 ± 0.09 b	80	2.42 ± 0.07 a	80	1.35 ± 0.05 d	77	2.04 ± 0.08 b
L3	74	1.79 ± 0.07 c	78	1.68 ± 0.06 c	56	1.82 ± 0.08 c	80	2.45 ± 0.07 a	80	2.01 ± 0.06 b	77	2.29 ± 0.07 a
L4	74	2.15 ± 0.06 b	77	1.75 ± 0.05 d	56	2.04 ± 0.05 b	79	3.40 ± 0.02 a	80	1.89 ± 0.04 c	77	2.04 ± 0.06 b
L5	73	2.48 ± 0.10 c	76	2.06 ± 0.04 d	56	2.09 ± 0.05 d	77	3.31 ± 0.12 a	80	2.50 ± 0.04 c	76	2.84 ± 0.11 b
L6 + L7	68	4.38 ± 0.14 b	64	3.56 ± 0.11 c	56	3.27 ± 0.13 c	72	4.66 ± 0.19 ab	77	4.27 ± 0.10 b	67	4.98 ± 0.16 a
Prepupa	59	1.22 ± 0.05 b	60	1.20 ± 0.05 b	56	1.70 ± 0.07 a	66	1.26 ± 0.06 b	77	1.00 ± 0.00 c	67	1.28 ± 0.05 b
Pupa	53	8.26 ± 0.12 b	46	8.85 ± 0.11 a	48	7.94 ± 0.11 c	47	9.08 ± 0.11 a	68	8.28 ± 0.09 b	62	8.37 ± 0.08 b
Preadult	53	27.24 ± 0.30 c	46	25.48 ± 0.26 d	48	26.65 ± 0.23 c	47	31.13 ± 0.33 a	68	26.60 ± 0.15 c	62	29.32 ± 0.21 b
**Adult**												
Female	25	10.00 ± 0.83b	20	5.65 ± 1.17 c	28	8.71 ± 0.63 b	21	12.47 ± 0.92 a	42	13.28 ± 0.83 a	30	11.53 ± 0.93 ab
Male	28	7.18 ± 0.67 bc	26	5.38 ± 0.93 c	20	6.35 ± 0.61 bc	26	9.15 ± 0.81 ab	26	10.61 ± 0.89 a	32	10.13 ± 0.87 a

Standard errors were estimated using 100,000 bootstrap resampling. Data followed by the same lower-case letter within a row were not significantly different based on a paired bootstrap test at 5% significance level.

**Figure 3:**
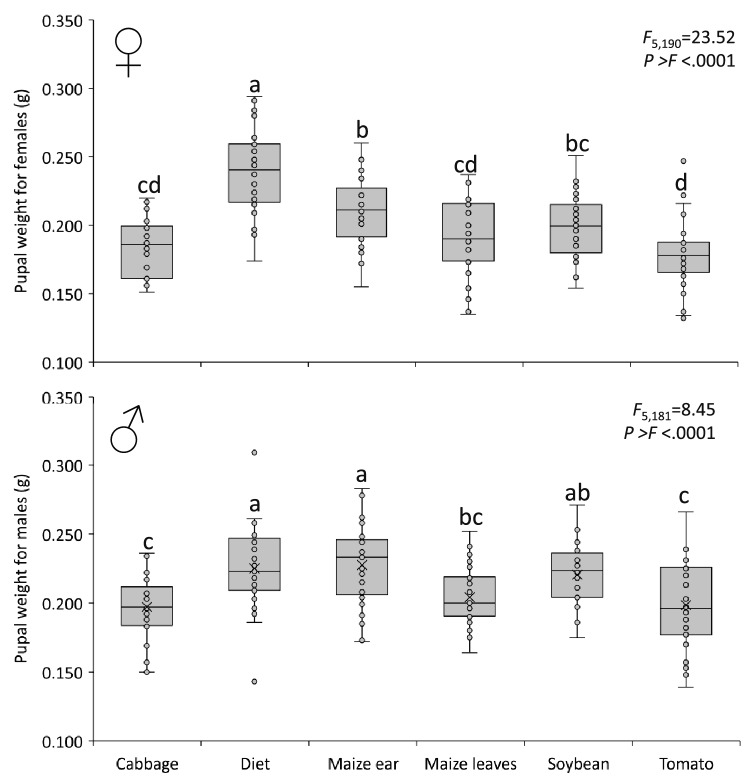
Pupal weight (mean ± SE) for females (top) and males (bottom) of the fall armyworm, *Spodoptera frugiperda* fed on cabbage, maize ear, maize leaves, artificial diet, soybean, and tomato.

Pre-adult stage duration was significantly shorter in maize ear, compared to artificial diet treatment, and followed by other vegetable treatments with intermediate length in the pre-adult stage duration (Table 2, Fig. 2). The highest percentage of survival was observed in soybean-reared insects (85%), followed by maize leaves (80%), tomato (77.5%), cabbage (66%), artificial diet (58.7%), and maize ear (57.5%) (Table 2). In line with this, the highest percentage of resulting females were observed in soybean (62% of pre-adults), followed by maize leaves (58%), tomato (48%), artificial diet (45%), maize ear (43%), and cabbage (38%) (Table 2, Fig. 2). In addition to the duration of the different FAW stages, the effect of treatments was also assessed on the pre-pupal weight (Fig. 3). Female pupae reared on artificial diet treatment were significantly heavier than pupae reared on cabbage, maize leaves, and tomato (Fig. 3), whereas male pupae reared on maize ear and diet were significantly heavier than pupae reared on cabbage and tomato (Fig. 3).

Not all females were reproductively active, and this was observed among all treatments. Therefore, the pre-oviposition period (APOP), total pre-oviposition period (TPOP), oviposition period, and fecundity parameters were calculated based on the information gathered from the reproductive females and not for the total number of females recorded (Table 3). Moreover, for cabbage, only 11 (44%) out of the 25 initially recorded females produced eggs, whereas in the case of maize ear, maize leaves, artificial diet, soybean, and tomato, the reproductive females were 25, 68, 29, 55, and 60% out of the total amount of females recorded for each treatment, respectively (Table 3).

**Table 3:** Adult preoviposition period (APOP), total preoviposition period (TPOP), oviposition period, and fecundity parameters based on reproductive females of the fall armyworm, *Spodoptera frugiperda* fed on cabbage, maize ear, maize leaves, artificial diet, soybean, and tomato.

Rearing media	Total females	Reproductive females	APOP (days)	TPOP (days)	Oviposition period (days)	Fecundity (viable eggs)
Cabbage	25	11	2.7 ± 0.6b	28.8 ± 0.8c	5.8 ± 1.05ab	1242.1 ± 204.3a
Diet	21	6	2.5 ± 0.6b	32.2 ± 1.3ab	4.0 ± 0.74bc	338.5 ± 90.8b
Maize ear	20	5	2.8 ± 0.8b	27.2 ± 1.1c	3.2 ± 0.67cd	803.2 ± 273.0ab
Maize leaves	28	19	5.1 ± 0.6a	31.4 ± 0.6a	2.2 ± 0.19d	278.1 ± 41.9b
Soybean	42	23	3.7 ± 0.5ab	29.6 ± 0.6bc	6.7 ± 0.51a	1006.5 ± 110.6a
Tomato	30	18	2.6 ± 0.3b	30.9 ± 0.5ab	4.9 ± 0.48b	842.3 ± 76.7a

Standard errors were estimated using 100,000 bootstrap resampling. Data followed by the same lower-case letter within a column were not significantly different based on a paired bootstrap test at 5% significance level.

**Figure 4:**
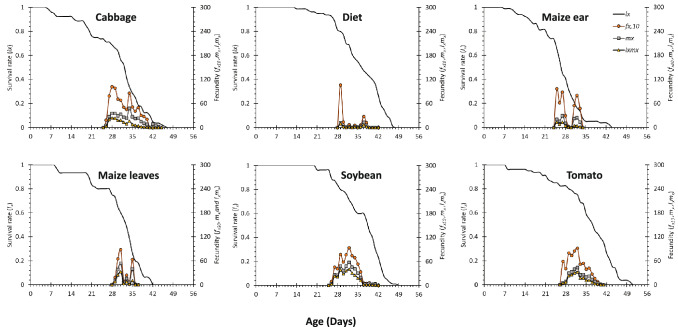
Age-specific survival rate (*l*_*x*_), fecundity (*m*_*x*_), and net maternity (*l*_*x*_*m*_*x*_) of the fall Armyworm, *Spodoptera frugiperda* fed on cabbage, maize ear, maize leaves, artificial diet, soybean, and tomato.

Feeding on different host plants also affected other parameters, including adult APOP, TPOP, oviposition period, and fecundity of the Fall armyworm (Table 3, Fig. 4). Females had the shortest APOP (i.e., number of days before a female start laying eggs and counted from adult emergence) in cabbage, artificial diet, maize ear, and tomato (2.5–2.6 days), whereas females reared in maize leaves started laying their eggs 5 days after emergence. In line with this, the TPOP (i.e., number of days that includes the pre-adult stage + APOP) was 4 days shorter in females fed on cabbage, maize ear, and soybean, compared to females reared on maize leaves (Table 3, Fig. 4). FAW females reared on soybean and cabbage had the longest oviposition period (i.e., the number of days that female produced offspring), almost double compared to maize treatments. In addition, females reared on cabbage, soybean, tomato, and maize ear laid 2.4–4.5 times more viable eggs compared to females reared on maize leaves and diet treatments (Table 3, Fig. 4).

**Figure 5:**
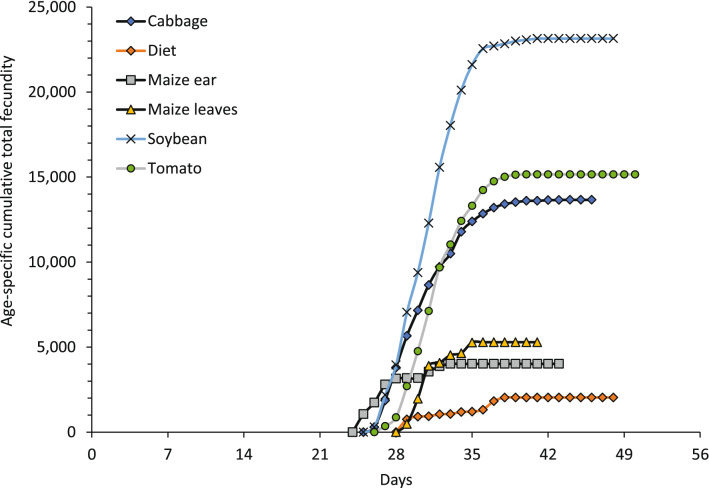
Cumulative fecundity of the fall Armyworm, *Spodoptera frugiperda* fed on cabbage, maize ear, maize leaves, artificial diet, soybean, and tomato.

The age‐specific survival rate (*l*_*x*_), fecundity (*m*_*x*_), and maternity (*l*_*x*_*m*_*x*_) values of the FAW reared on six different treatments are plotted in Fig. 4. Highest peaks on fecundity were observed for females fed and ovipositing on cabbage, soybean, tomato, followed by artificial diet and maize with the lowest fecundity values. Maternity curves (*l*_*x*_*m*_*x*_) had a higher peak on soybean, tomato, and cabbage, followed by lower curves in maize and artificial diet treatments. In addition, information on cumulative fecundity including all reproductive females in each treatment showed a higher total contribution of viable eggs (i.e. larvae) in soybean, cabbage and tomato treatments, compared to maize treatments (Table 3, Fig. 5).

Net reproductive rate (*R*_0_) was 5.7 and 11.4 times bigger in soybean compared to maize ear and artificial diet, respectively (Table 4). The intrinsic rate of increase (*r*) and finite rate of increase (*λ*) of FAW were higher on soybean, tomato, and cabbage compared to maize (intermediate), and artificial diet (lowest), which suggest FAW populations will grow faster in the vegetable crops tested compared to the artificial diet. In contrast, a shorter mean generation time was observed in females from the maize treatment compared to the other vegetable crops tested.

**Table 4:** Mean (± SE) of the net reproductive rate (*R*_0_), intrinsic rate of increase (*r*), finite rate (*λ*), and mean generation time (*T*) of the fall armyworm, *Spodoptera frugiperda* fed on cabbage, maize ear, maize leaves, artificial diet, soybean, and tomato.

Treatment	*R*_0_ (offspring/individual)	*r* (day^-1^)	*λ* (day^-1^)	*T* (day)
Cabbage	170.79 ± 54.75 a	0.1651 ± 0.0115 ab	1.1795 ± 0.0135 ab	31.14 ± 0.76 ab
Maize ear	50.20 ± 26.19 bc	0.1397 ± 0.0362 abc	1.1499 ± 0.0383 abc	28.02 ± 1.27 b
Maize leaves	88.07 ± 21.14 b	0.1395 ± 0.0083 b	1.1497 ± 0.0095 b	32.10 ± 0.35 a
Diet	25.39 ± 11.66 c	0.0970 ± 0.0207 c	1.1020 ± 0.0221 c	33.32 ± 1.88 a
Soybean	289.36 ± 59.70 a	0.1791 ± 0.0075 a	1.1962 ± 0.0089 a	31.64 ± 0.43 a
Tomato	189.52 ± 42.80 a	0.1615 ± 0.0081 ab	1.1752 ± 0.0095 ab	32.48 ± 0.54 a

Standard errors were estimated using 100,000 bootstrap resampling. Data followed by the same lower-case letter within a column were not significantly different based on a paired bootstrap test at 5% significance level.

In this study, we also did a general assessment of the nutritional quality of the treatments tested (Table 5), and we found that the amount of protein and nitrogen contained in the artificial diet treatment was 5- and 10-fold higher compared to soybean, tomato, cabbage, and maize, whereas the amount of fat was 2–5 higher for the same treatments compared to diet. In the case of chlorogenic acid, the amount contained in tomato was 3- and 10-fold higher compared to soybean and cabbage, respectively, and this compound was not detected in maize and artificial diet. Caffeic acid, and quercetin were not detected in soybean, maize and artificial diet, whereas the amount in tomato was 2- and 10-fold higher compared to cabbage, respectively.

**Table 5:** Nutritional value analysis of different rearing media based on 100 g edible portion on fresh weight.

Treatment	DM (g)	Protein (g)	Nitrogen (g)	Oil (g)	Chlorogenic acid (μmole)	Caffeic acid (μmole)	Quercetin (μmole)	Kaempferol (μmole)	Phenolics (mg)
Cabbage leaves	11.3	0.42	0.07	0.47	3.22	18.71	1.39	27.78	272
Tomato leaves	13.4	0.70	0.11	0.66	32.05	36.78	12.11	4.88	310
Soybean leaves	19.4	0.85	0.14	0.73	8.48	0.00	0.00	189.60	393
Maize	21.1	0.41	0.06	1.25	0.00	0.00	0.00	0.00	93
Diet (dry powder)	90.8	4.04	0.65	2.57	0.00	0.00	0.00	0.00	2933

(*DM* dry matter). Luteolin, apigenin, isohamnetin, hesperetin were not found in the processed samples.

## Discussion

In this study, we investigated the ovipositional preference of FAW female moths on different host plants, under two experimental arenas, choice test and no-choice test. Moreover, a masking odor effect was also investigated using the most and the least preferred host plants. Later, using the age-stage two-sex life table theory^27,28^ we assessed the performance of immature FAW individuals fed and reared on cabbage, maize (ear and leaves), soybean, tomato, and an artificial diet, in order to get information related to development time, survival, reproduction and longevity of FAW.

As we expected, FAW females had an oviposition preference for maize compared to other vegetable crops, including cabbage and soybean. However, we did not expect FAW reluctance for tomato. Therefore, the no-choice test was performed in order to confirm the preference or avoidance of the most and least preferred host plants, maize and tomato, respectively. FAW laid similar amount of egg masses on the inner walls of the cage as well as in the maize plants, but, in contrast, FAW highly preferred the cage wall to lay their eggs instead of the tomato plants. Oviposition on non-plant material (i.e., cage walls) despite the presence of host plants has been previously recorded for FAW^29,30^. More specifically, Rojas et al.^29^ found strong tactile stimuli for grooved and pitted surfaces on which FAW oviposit regardless the presence of chemical cues. Similar observation has been recorded for the generalist moths, *Spodoptera exigua*^31^, and *Epiphyas postvittana* (Tortricidae)^32^, where tactile stimuli seem to be as important as the chemical cues in terms of ovipositional behavior and preference.

When the masking effect was estimated, FAW females still preferred to lay their eggs in the cage wall compared to the tomato sprayed with maize extract, suggesting tomato plants were still avoided, regardless of the additional olfactory/gustatorial cues provided by the maize extract. It is also possible that the most important oviposition stimulant(s) from maize may not have been present in the extract, which was sprayed on to tomato plants. It should be noted that FAW female moths laid more eggs on control areas than on substrates treated with leaf extracts of maize. Therefore, tomato plants were not favored by maize extract. On the other hand, when maize plants were sprayed with tomato extract, FAW were not deterred and the oviposition did not decrease. An earlier study also confirmed that substrates treated with tomato leaf extracts were less preferred for egg laying by FAW female moths^29^. This result suggests that all antixenotic factors from tomato (i.e., including trichomes and chemical exudates from them) were not present in the extract to deter FAW from choosing maize plants as ovipositional substrate. It was also found that ovipositional deterrents were present in tomato plants^29^.

**Figure 6:**
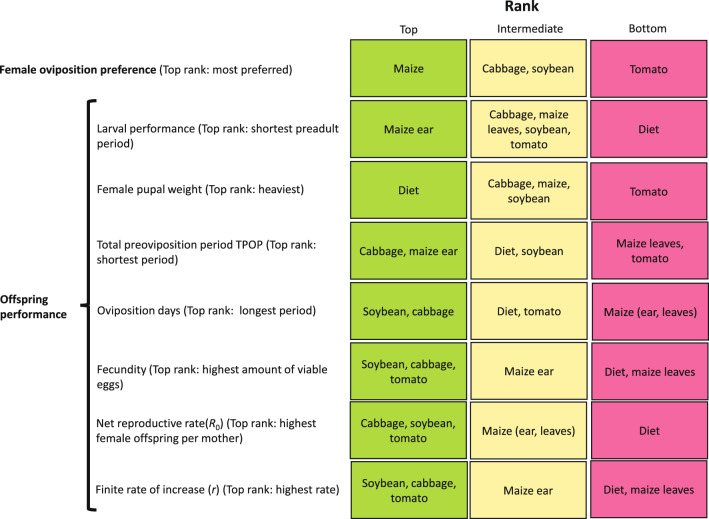
Summary of the preference and performance of the fall armyworm, *Spodoptera frugiperda* fed on cabbage, maize ear, maize leaves, artificial diet, soybean, and tomato.

Based on the preference-performance hypothesis^22,23^, this part of our study suggests that female moths choose to oviposit in hosts with the highest nutritional quality for offspring, leading to shorter developmental time, increased biomass, and higher reproductive potential in FAW offspring reared on maize. Moreover, it is expected that the ovipositional preference of FAW female moths would align with the performance of their offspring. Thus, we assumed that FAW female moths reared on maize plants (ears and leaves) would have decreased developmental time, prolonged ovipositional period, and increased reproductive potential, which were demonstrated not only in terms of fecundity numbers, but also in terms of positive impact on reproductive parameters, including higher net reproductive rate (*R*_0_), intrinsic rate of increase (*r*) and finite rate of increase (*λ*). However, our results did not completely align with the preference-performance hypothesis. First, in terms of gained biomass, pupae reared on diet treatment were heavier than those reared on other vegetable crops. Second, in terms of reproductive potential and parameters, we got mixed results, with interesting outcome in terms of total reproductive females (i.e., egg producing females) observed in maize (i.e., ear and leaves), accounting between 25 and 68% of the total females reared on those treatments. Moreover, FAW females reared on soybean and cabbage almost doubled their oviposition period compared to maize plants. In addition, females reared on cabbage, soybean, tomato, and maize ear laid 4–6 times more viable eggs compared to females reared on maize leaves and artificial diet treatments. The higher number of reproductive females reared on the vegetable crop treatments would suggest in theory, a significant population growth in these crops under field conditions. However, and although the life table studies were carried out simultaneously and under controlled conditions, in the future it is also necessary to confirm these results under field and free choice conditions. Second, in terms of reproductive parameters, the net reproductive rate (*R*_0_) was higher in soybean compared to maize, whereas the intrinsic rate of increase (*r*) and finite rate of increase (*λ*) of FAW were higher on vegetable crops compared to maize (intermediate), and diet (lowest). The evaluated ecological parameters suggest that FAW populations grow faster in the vegetable crops compared to maize. Furthermore, results from this study suggest that cabbage, soybean, and tomato seemed to provide a high benefit for an appropriate offspring performance, exceeding in some cases the benefits from its usual maize-based diet (Fig. 6).

In a previous study, the host preference by female moths and offspring performance were documented for the FAW by comparing the discrimination among three maize cultivars with different degrees of resistance^33^. In a choice test, it was found that moths laid more eggs on the highly resistant cultivar and least on the susceptible cultivar, whereas larval performance was poor in the former but good in the susceptible. In addition, the same study found that older FAW larvae did not show any discrimination or preference among the tested maize cultivars, nor even for real versus model maize plants, suggesting larvae may have a random foraging behavior independent of volatile cues, with low discrimination among hosts and based on visual cues. An earlier study also confirmed that plant volatiles did not influence host finding by FAW, but tactile cues may be of important during the selection of oviposition sites^29^.

In contrast, a recent paper using the age-stage two-sex life table and adult female oviposition preference for the FAW^30^ found consistent results with the preference performance hypothesis on FAW, when comparing the larval performance and oviposition preference of the FAW among maize, potato and tobacco. More specifically, the FAW reared on maize had the strongest development performance (i.e. shortest development time, prolonged longevity and higher reproductive rate) compared to potato (intermediate), and tobacco (least suitable host). More recently, another paper comparing the fitness of the FAW on three solanaceous crops, found that FAW was able to complete the life cycle on pepper and tomato, with exception of eggplant. Moreover, the authors also found that FAW larvae reared on tomato had a better fitness in terms of development of duration, survival rate, fecundity, and *R*_0_ based on the developmental duration, compared to maize^34^.

In all, a random foraging behavior that seems to be independent of volatile cues but linked to a strong tactile stimulus seems to be driving the ovipositional behavior and preference for specific host plants. However, in terms of larval development and performance, the fitness of the resulting FAW progeny is not necessarily align with the mother’s choice, and therefore, results cannot be explained under a preference performance hypothesis.

In terms of nutritional aspects and differences among treatments, chlorogenic acid, which was present in tomato and cabbage, is an antioxidant polyphenol, a conjugate of caffeic acid and quinic acid, and is recognized as an efficient defense molecule against several insect herbivores, including *Helicoverpa armigera*, *Spodoptera litura*, *Spodoptera exigua*, thrips, and leaf beetles^35,36^, since this compound reduce the availability of amino acids and in consequence decreases the digestibility of dietary proteins, leading to a lower nutritional value, and thus resulting in growth reduction^36–38^. In addition, the presence of chlorogenic acid in tomato and cabbage would partially explain the low weight of female pupae on these treatments^39^.

In contrast, previous information suggested that despite the defensive compounds such as glycoalkaloids, phenolics and terpenes, as well as other anti-nutritive proteins^40^ present in tomato, FAW was reported to feed and successfully complete its life cycle on tomato leaves^41^. However, a more recent study looking at the alterations to flight muscle protein structure and flight-related fitness traits of two armyworms caused by host plant defenses^42^ suggested that FAW seems to show some degree of specialization for monocot crops (grasses), since its average survival was lower when feeding on tomato compared to a related species, the southern armyworm (SAW), *Spodoptera eridania*, a species that feeds on leafy dicots. The study also reported that FAW was less adapted than SAW to feed on tomato and cope with its defensive secondary metabolites.

Regarding other metabolites found in this study, the effect of flavonoids on insects has been extensively discussed. The mortality of the tobacco armyworm (*S. litura*) was previously linked to quercetin present on peanuts^43^. In addition, kaempferol and quercetin among other flavonols have been studied for their deterrent activity against the nematodes *Radopholus similis* and *Meloidogyne incognita* and for their anti-pathogenic activities on different cereal and vegetable crops [^44^ and references therein]. Furthermore, previous studies had suggested that a reduction of various flavonoids, including kaempferol and quercetin metabolites may facilitate the puncturing of the symplast and favoring a subsequent aggregation of the whitefly, *Bemisia tabaci*^45^. However, given that our scope in this study is not directly related to the nutritional content and differences among plants, a more detailed study would be needed to properly provide evidence in how the presence of these nutritional/anti-nutritional/defensive compounds directly affect the performance of the feeding larvae and the impact on their reproductive fitness.

From an insect pest management perspective, our results are relevant because understanding the discrimination of FAW female moths for suitable hosts for oviposition vs. larval performance would give us a better idea of the risk for vegetable crops located in the vicinity of maize crops in newly invaded geographical regions. High and random dispersal of larvae would potentially move to alternate hosts to complete development. In addition, the above-mentioned vegetables may serve as appropriate host to improve the reproductive fitness of the next generations, since reproductive parameters would be positively impacted compared to the benefits offered from a maize-based diet. Therefore, the use of appropriate crop rotations in the vicinity of maize crops must consider the potential damage and the need to use alternative strategies, like the use of resistant cultivars or appropriate trap crops. A similar concern has been also raised in terms of intercropping systems with maize, potato, tobacco, pepper, and tomato^30,34^ since late instars of the FAW would be able to move to less preferred host plants after consuming maize, posing a serious threat to less preferred crops, but on which FAW would be capable of completing its development. At the moment, more information needs to be gathered in Taiwan to assess the risk and potential economic damage of FAW on vegetable crops.

Lastly, it is also important to discuss our results in terms of the potential threats of the invasive FAW under island conditions. As pointed out by Kairo et al.^46^ and Mochizuki^47^, the greatest threat to island biodiversity and habitat loss is the growing problem of invasive species. Hence, the arrival of the FAW to Taiwan would potentially affect the niche spaces and the competition with other insect pests on the area, but also, the lack of natural enemies will provide a significant advantage for its establishment, and in addition, adaptive changes to particular climatic conditions plus isolation may provide further opportunities for the emergence of additional FAW populations, which could also expand its host range including dicot crops such as vegetables. Furthermore, economically important insect pests including the FAW are already resistant to chemical pesticides in regions of original distribution, so conventional chemical control of this insect pest may become difficult in newly invaded areas. Therefore, there is a high priority to work with integrated pest management strategies that allow the use of alternative safe control strategies to keep FAW numbers low.

## Conclusions

Overall, our results suggest that despite low preference of FAW females for vegetable crops, these crops seemed to provide a high benefit for an appropriate offspring performance, exceeding in some cases the benefits from its usual maize-based diet. In that sense, the reproductive fitness of the next generations may be more positively impacted compared to the benefits offered from a maize-based diet, suggesting the need to consider appropriate crop rotations and other sustainable management strategies in the vicinity of maize crops in order to reduce the potential damage.

## Methods

### Location

The experiments were conducted at the World Vegetable Center, Shanhua, Tainan, Taiwan (23° 08′ 29″ N, 120° 19′ 15″ E) at a mean elevation of 9 m above the sea level and due to the invasive nature of the fall armyworm, all observations were recorded under quarantine facilities.

### Host plants

Commercially available cabbage (Brassicaceae: *Brassica oleracea* var. capitata, ‘early autumn’, TAKII & Co., Ltd., Kyoto, Japan), maize (Poaceae: *Zea mays*, ‘Tienmimi’, HO-HUAN Agricultural Product Co., Ltd., Nantou, Taiwan), soybean (Fabaceae: *Glycine max*, ‘Kaohsiung No. 9’, Kaohsiung District Agricultural Research and Extension Station DARES, Kaohsiung, Taiwan), and tomato (Solanaceae: *Solanum lycopersicum*, ‘Taoyuan Yasu No. 20’, Taiwan Seed Improvement and Propagation Station, Taoyuan, Taiwan) seedlings were sown in seedling trays filled with culture media (King Root ® No. 1, Dayi Agritech Co. Ltd., Taipei, Taiwan) and maintained under greenhouse conditions at the World Vegetable Center, Taiwan, and maintained under identical growing conditions (26 ± 1 °C, 70 ± 10% RH, photoperiod of 14:10 [L:D] h). Pots were watered as needed, and every 7–10 days, a foliar granular fertilizer (N:P:K = 12:18:12, #39 Biotec Organic Compound Fertilizer, Taiwan Fertilizer Co. Ltd, Taipei, Taiwan) was applied with 0.5–4 g according to the plant stage. In addition to the vegetables tested, our control treatment included an artificial diet^48^. All plant experiments were conducted according to institutional guidelines.

### Fall armyworm rearing

Given the new presence of this pest in Taiwan, eggs and larvae of *S. frugiperda* were initially provided by the Taiwan Agricultural Research Institute (TARI), Council of Agriculture (COA) and maintained under controlled conditions [26 ± 1.5 °C, 50 ± 10%RH, 14:10 h photoperiod regime (L:D)] in quarantine facilities of World Vegetable Center, Taiwan. The number of eggs and resulting 1st instar larvae used for the different treatments is presented in Table 2.

### Oviposition preference: free choice test for fall armyworm females

To determine which plant species is preferred for oviposition, a preference test was conducted on cabbage, maize, soybean, and tomato. Four 1-month old seedlings of each crop were arranged inside a mesh cage (60 × 60 × 60 cm tent). The experimental arena consisted on four concentric circles formed by one seedling per crop. A total of 16 seedlings (four seedlings/crop/four concentric circles) were arranged in each mesh cage. A total of 70 FAW pairs were used for this experiment, with ten virgin pairs released at the center of each cage, for a total of seven replications. Female adults were allowed to oviposit for 6 days, and pure honey was provided ad-libitum as food resource for FAW adults. Egg masses and egg numbers were recorded on each crop and averaged within each cage after 6 days. Egg masses laid on the cage surface were discarded.

### Oviposition preference: no-choice test for fall armyworm females

A follow up experiment was conducted to confirm the oviposition preference of female adults with the least and most preferred host plants (i.e., tomato and maize). In this experiment, we used additional treatments to assess a potential odor masking of both plants, and how it may affect pest insects' ability to find hosts. In that sense, tomato plants coated with maize extract and maize plants coated with tomato aqueous extract were tested. For this, 100 g of tomato or maize leaves from 1.5 to 2-month-old seedlings were collected and mixed with 400 ml of water and then liquefied. The resulting aqueous extract was applied to the treated plants (i.e., maize and tomato, respectively) by coating all surfaces by dipping or brushing. The plants were allowed to dry for 4 h before the start of the experiment. A total of three seedlings per host plant were offered to four virgin pairs released at the center of each cage, for a total of 12 replications. Female adults were allowed to oviposit for 5 days, and pure honey was provided as ad-libitum as food resource for FAW adults. Egg masses and egg numbers were recorded on each crop and averaged within each cage after 6 days.

### Biological traits and demographic parameters of fall armyworm reared on different host plants

Due to the invasive status of this insect pest in Taiwan, the egg batches used in this study were derived from first generation of laboratory stocks from field caught FAW females from the initially eggs provided by the Taiwan Agricultural Research Institute (TARI), Council of Agriculture (COA) as previously mentioned. Furthermore, different biological traits including the development, survival, reproduction, and ecological parameters of FAW fed on cabbage, maize ear, maize leaves, soybean, tomato, and artificial diet were compared. Egg batches received from TARI were placed under controlled conditions and newly hatched larvae were individually placed and kept in plastic containers (sauce cup; 4 cm × 3 cm; 40 ml) and provided with fresh leaves of the selected crops (approx. 1–2 g or 30 cm^2^ of food) on a daily basis and until they reached pre-pupal stage. Cabbage, soybean, and tomato were given as fresh leaves, and in the case of maize, larvae were offered fresh leaves and maize ear. Each day, the development and survival of larvae were observed for each treatment. Larval stages were L1, L2, L3, L4, L5, and L6–7. Pupal weight was collected from the surviving insects, and sex determination was conducted at the pupal stage. Pupae were individually kept and after adult emergence, one female and one male were placed in plastic containers covered with plastic cups (drinking cup; 5.5 cm × 10 cm; 350 ml). Oviposition substrate used to collect the resulting eggs corresponded to the same treatment food they were offered as immatures. In the case of the artificial diets, resulting adults were offered with an unscented corrugated paper and adults from all treatments were fed with a 10% honey solution. Observations of insect biological parameters and development including duration of instars, sex, and longevity of individuals were recorded on a daily basis. In line with this, fecundity and survival data were recorded daily until the death of each individual and for each environmental condition tested.

### Nutritional quality

The nutritional quality of uninfested dry leaves of the tested vegetables was estimated through various biochemical analysis (modified), including dry matter^49^, crude fat^50^, total nitrogen^51^, flavonoids^52^, and total phenolics^53^.

### Statistical analysis

One way analysis of variance (ANOVA) and Tukey’s HSD post-hoc test were used to assess the oviposition preference and pupal weight of FAW among cabbage, maize, soybean, and tomato. For the second experiment (i.e. non-choice test), FAW egg masses and number of eggs recorded in the plants and the cage walls were compared by paired t-test. Differences were considered highly significant at P < 0.001; and significant at P < 0.05. Statistical analyses were performed using SAS software version 9.4 (SAS institute, Cary, NC, USA).

The raw data for each FAW individual within different treatments were analyzed using the age-stage two-sex life table theory^27,28^, on which daily raw data for developmental time, survival, and fecundity was recorded. The life history parameters, including the age-stage survival rate (*s*_*xj*_, where age = *x*, and stage = *j*), the age-specific survival rate (*l*_*x*_), age-specific fecundity (*m*_*x*_), and the population parameters (*R*_0_, net reproductive; *r*, intrinsic rate of increase; *λ*, finite rate of increase; *T*, mean generation time) were calculated using the computer program, TWOSEX-MSChart^54^. The age-specific survival rate, *l*_*x*_ (Eq. 1), which is the probability that a newly laid egg will survive to age *x*, was calculated as:

1 $$l_{x} = \sum\limits_{j = 1}^{\delta } {s_{xj} }$$

where, *δ* is the last stage of the study cohort.

The age-specific fecundity of the population, *m*_*x*_ (Eq. 2) was calculated as:

2 $$m_{x} = \frac{{\sum\nolimits_{j = 1}^{\delta } {s_{xj} f_{xj} } }}{{\sum\nolimits_{j = 1}^{\delta } {s_{xj} } }}$$

The net reproductive rate, *R*_0_ (Eq. 3), which represents the mean number of offspring that an individual can produce during its lifetime, was calculated as:

3 $${R}_{0}={\sum }_{x=0}^{\infty }{l}_{x}{m}_{x}$$

The intrinsic rate of increase, *r* (Eq. 4) was estimated using the iterative bisection method from the Euler-Lotka formula with age indexed from 0^55^:

4 $$\sum_{x=0}^{\infty }{e}^{-r\left(x+1\right)}{l}_{x}{m}_{x}=1$$

The finite rate of increase, λ (Eq. 5), and the mean generation time, T were then calculated as follows:

5 $$\lambda = {e}^{r}$$

6 $$T=\frac{{\text{ln}R}_{0}}{r}$$

The variances and standard errors of the population parameters and life history parameters, including developmental time, adult longevity, and fecundity were calculated with the bootstrap technique^56–60^ with 100,000 random resampling. Significant difference at P < 0.05 among different treatments were compared with Paired bootstrap test implemented in TWOSEX-MSChart^61–63^. Fecundity was measured in terms of viable eggs and unhatched eggs were not considered for our demographic analysis^64^.

### Ethical standards

This research did not involve any human participants and/or animals, other than the fall armyworm, *S. frugiperda*.

## References

[1] Goergen G, Kumar PL, Sankung SB, Togola A, Tamò M (2016). First report of outbreaks of the fall armyworm *Spodoptera frugiperda* (J E Smith) (Lepidoptera, Noctuidae), a new alien invasive pest in West and Central Africa. PLoS ONE.

[2] IITA News. First report of outbreaks of the "Fall Armyworm" on the African continent. *IITA Bulletin* **2330**. http://bulletin.iita.org/index.php/2016/06/18/first-report-of-outbreaks-of-the-fall-armyworm-on-the-african-continent/ (2016).

[3] FAO. Briefing note on FAO actions on fall armyworm. Policy brief/paper. http://www.fao.org/3/bs183e/bs183e.pdf (2019).

[4] Vennila, S. *et al.* G20 discussion group on fall armyworm *Spodoptera frugiperda* (J.E.Smith) [Lepidoptera: Noctuidae]. In *Proceedings of the International Workshop on Facilitating International Research Collaboration on Transboundary Plant Pests, Tsukuba, Japan*. https://www.affrc.maff.go.jp/kokusaikenkyu/transboundary_plant_pests_e_2019.html (2019).

[5] OEPP/EPPO. EPPO Global Database. *Spodoptera frugiperda*. EPPO Standards. EPPO A1 and A2 list of pest recommended for regulation as quarantine pests, PM 1/2(29) English. https://gd.eppo.int (2020).

[6] Ma J (2019). High risk of the fall armyworm invading into Japan and the Korean Peninsula via overseas migration. J. Appl. Entomol..

[7] Westbrook JK, Nagoshi RN, Meagher RL, Fleischer SJ, Jairam S (2016). Modeling seasonal migration of fall armyworm moths. Int. J. Biometeorol..

[8] Cock MJW, Beseh PK, Buddie AG, Cafá G, Crozier J (2017). Molecular methods to detect *Spodoptera frugiperda* in Ghana, and implications for monitoring the spread of invasive species in developing countries. Sci. Rep..

[9] Du Plessis H, Schlemmer ML, Van den Berg J (2020). The effect of temperature on the development of *Spodoptera frugiperda* (Lepidoptera: Noctuidae). Insects.

[10] Nagoshi NR, Meagher RL, Hay-Roe M (2012). Inferring the annual migration patterns of fall armyworm (Lepidoptera: Noctuidae) in the United States from mitochondrial haplotypes. Ecol. Evol..

[11] Early R, González-Moreno P, Murphy ST, Day R (2018). Forecasting the global extent of invasion of the cereal pest *Spodoptera frugiperda*, the fall armyworm. NeoBiota.

[12] Zacarias DA (2020). Global bioclimatic suitability for the fall armyworm, *Spodoptera frugiperda* (Lepidoptera: Noctuidae), and potential co-occurrence with major host crops under climate change scenarios. Clim. Change.

[13] Hruska AJ, Gould F (1997). Fall armyworm (Lepidoptera: Noctuidae) and *Diatraea lineolata* (Lepidoptera: Pyralidae): Impact of larval population level and temporal occurrence on maize yield in Nicaragua. J. Econ. Entomol..

[14] Day R (2017). Fall armyworm: Impacts and implications for Africa. Outlooks Pest Manag..

[15] FAO. Forecasting threats to the food chain affecting food security in countries and regions. Food Chain Crisis Early Warning Bulletin. No. 36, July–September 2020. Rome. http://www.fao.org/3/cb0160en/cb0160en.pdf (2020).

[16] Montezano DG (2018). Host plants of *Spodoptera frugiperda* (Lepidoptera: Noctuidae) in the Americas. Afr. Entomol..

[17] Nagoshi RN (2018). Analysis of strain distribution, migratory potential, and invasion history of fall armyworm populations in northern Sub-Saharan Africa. Sci. Rep..

[18] Tsai CL (2020). Rapid identification of the invasive fall armyworm *Spodoptera frugiperda* (Lepidoptera, Noctuidae) using species-specific primers in multiplex PCR. Sci. Rep..

[19] Haenniger S (2020). Sexual communication of *Spodoptera frugiperda* from West Africa: Adaptation of an invasive species and implications for pest management. Sci. Rep..

[20] Nagoshi RN (2019). Evidence that a major subpopulation of fall armyworm found in the Western Hemisphere is rare or absent in Africa, which may limit the range of crops at risk of infestation. PLoS ONE.

[21] Zhang L (2020). Genetic structure and insecticide resistance characteristics of fall armyworm populations invading China. Mol. Ecol. Res..

[22] Pӧykkӧ H (2006). Females and larvae of a geometrid moth, *Cleorodes lichenaria*, prefer a lichen host that assures shortest larval period. Environ. Entomol..

[23] Refsnider JM, Janzen FJ (2010). Putting eggs in one basket: Ecological and evolutionary hypotheses for variation in oviposition-site choice. Annu. Rev. Ecol. Evol. Syst..

[24] Thompson JN (1988). Evolutionary ecology of the relationship between oviposition preference and performance of offspring in phytophagous insects. Entomol. Exp. Appl..

[25] Nylin S, Janz N (1996). Host plant preferences in the comma butterfly (*Polygonia c-album*): Do parents and offspring agree?. Ecoscience.

[26] Janz N, Nylin S (1997). The role of female search behavior in determining host plant range in plant feeding insects: A test of the information processing hypothesis. Proc. Biol. Sci..

[27] Chi H, Liu H (1985). Two new methods for the study of insect population ecology. Bull. Inst. Zool. Acad. Sin..

[28] Chi H (1988). Life-table analysis incorporating both sexes and variable development rates among individuals. Environ. Entomol..

[29] Rojas JC, Virgen A, Cruz-López L (2003). Chemical and tactile cues influencing oviposition of a generalist moth, *Spodoptera frugiperda* (Lepidoptera: Noctuidae). Environ. Entomol..

[30] Guo JF (2020). Comparison of larval performance and oviposition preference of *Spodoptera frugiperda* among three host plants: Potential risks to potato and tobacco crops. Insect Sci..

[31] Greenberg SM, Sappington TW, Sétamou M, Liu TX (2002). Beet armyworm (Lepidoptera: Noctuidae) host plant preferences for oviposition. Env. Entomol..

[32] Foster SP, Howard AJ, Harris MO (1997). The influence of tactile and other non-chemical factors on the ovipositional responses of the generalist herbivore *Epiphyas postvittana*. Entomol. Exp. Appl..

[33] Rojas JC, Kolomiets MV, Bernal JS (2018). Nonsensical choices? Fall armyworm moths choose seemingly best or worst hosts for their larvae, but neonate larvae make their own choices. PLoS ONE.

[34] Wu LH (2021). Fitness of fall armyworm, *Spodoptera frugiperda* to three solanaceous vegetables. J. Integr. Agric.

[35] Bhonwong A, Stout MJ, Attajarusit J, Tantasawat P (2009). Defensive role of tomato polyphenol oxidases against cotton bollworm (*Helicoverpa armigera*) and beet armyworm (*Spodoptera exigua*). J. Chem. Ecol..

[36] Kundu A, Vadassery J (2019). Chlorogenic acid-mediated chemical defense of plants against insect herbivores. Plant Biol. (Stuttg.).

[37] Felton GW, Duffey SS (1991). Protective action of midgut catalase in lepidopteran larvae against oxidative plant defenses. J. Chem. Ecol..

[38] Leiss KA, Maltese F, Choi YH, Verpoorte R, Klinkhamer PGL (2009). Identification of chlorogenic acid as a resistance factor for thrips in chrysanthemum. Plant Physiol..

[39] Niggeweg R, Michael AJ, Martin C (2004). Engineering plants with increased levels of the antioxidant chlorogenic acid. Nat. Biotechnol..

[40] Schilmiller AL, Howe GA (2005). Systemic signaling in the wound response. Curr. Opin. Plant Biol..

[41] Capinera JL (2001). Handbook of Vegetable Pests.

[42] Portman SL, Felton GW, Kariyat RR, Marden JH (2020). Host plant defense produces species-specific alterations to flight muscle protein structure and flight-related fitness traits of two armyworms. J. Exp. Biol..

[43] Mallikarjuna N, Kranthi KR, Jadhav DR, Kranthi S, Chandra S (2004). Influence of foliar chemical compounds on the development of *Spodoptera litura* in interspecific derivatives of groundnut. J. Appl. Entomol..

[44] Mierziak J, Kostyn K, Kulma A (2014). Flavonoids as important molecules of plant interactions with the environment. Molecules (Basel, Switzerland).

[45] Su Q (2018). Whitefly aggregation on tomato is mediated by feeding-induced changes in plant metabolites that influence the behavior and performance of conspecifics. Funct. Ecol..

[46] Kairo, M., Ali, B., Cheesman, O., Haysom, K., & Murphy, S. Invasive Species Threats in the Caribbean Region. Report to the Nature Conservancy. (CAB International, 2003)

[47] Mochizuki A (2008). Invasive Insects: Problem and Risk Assessment.

[48] Ku, C.I. Pathogenicity of indigenous nematode *Steinernema* sp. (isolate 39) against *Helicoverpa armigera* (Lepidoptera: Noctuidae) and its potential application. Master thesis, National Chung Hsing University, Taiwan. P1–64 (2016).

[49] AOAC. Official methods of analysis: Official method 934.01, moisture in animal feed. in *Official Methods of the Association of Official Analytical Chemists* (1990).

[50] AOAC. Official methods of analysis: Official method 948.22, fat (crude) in nuts and nut products. in *Official Methods of the Association of Official Analytical Chemists* (1990).

[51] AOAC. Official methods of analysis: Official method 978.04, crude protein in plants. in *Official Methods of the Association of Official Analytical Chemists* (1990).

[52] Yang RY, Lin S, Kuo G (2008). Content and distribution of flavonoids among 91 edible plant species. Asia Pac. J. Clin. Nutri..

[53] Singleton VL, Rossi JA (1965). Colorimetry of total phenolics with phosphomolybdic-phosphotungstic acid reagents. Am. J. Enol. Viticult..

[54] Chi, H. TWOSEX-MSChart: A computer program for the age-stage, two-sex life table analysis. http://140.120.197.173/Ecology/ (2021).

[55] Goodman D (1982). Optimal life histories, optimal notation, and the value of reproductive value. Am. Nat..

[56] Efron B, Tibshirani R (1993). An Introduction to the Bootstrap.

[57] Huang YB, Chi H (2012). Assessing the application of the jackknife and bootstrap techniques to the estimation of the variability of the net reproductive rate and gross reproductive rate: a case study in *Bactrocera cucurbitae* (Coquillett) (Diptera: Tephritidae). J. Agric. For..

[58] Huang YB, Chi H (2012). Life tables of *Bactrocera cucurbitae* (Diptera: Tephritidae): With an invalidation of the jackknife technique. J. Appl. Entomol..

[59] Huang YB, Chi H (2012). Age-stage, two-sex life tables of *Bactrocera cucurbitae* (Coquillett) (Diptera: Tephritidae) with a discussion on the problem of applying female age-specific life tables to insect populations. Insect Sci..

[60] Chi H (2020). Age-stage, two-sex life table: An introduction to theory, data analysis, and application. Entomol. Gen..

[61] HesHesterberg T, Moore DS, Monaghan S, Clipson A, Epstein R, Moore DS, McCabe GP (2005). Bootstrap methods and permutation tests. Introduction to the Practice of Statistics.

[62] Smucker, M. D., Allan, J. & Carterette, B. A. A comparison of statistical significance tests for information retrieval evaluation. IN *CIKM '07: Proceedings of the sixteenth ACM conference on Conference on information and knowledge management, New York, NY, USA*, pp. 623–632. (2007).

[63] Wei MF (2020). Demography of *Cacopsylla chinensis* (Hemiptera: Psyllidae) reared on four cultivars of *Pyrus bretschneideri* and *P. communis* (Rosales: Rosaceae) pears with estimations of confidence intervals of specific life table statistics. J. Econ. Entomol..

[64] Mou DF, Lee CC, Smith CL, Chi H (2015). Using viable eggs to accurately determine the demographic and predation potential of *Harmonia dimidiata* (Coleoptera: Coccinellidae). J. Appl. Entomol..

