# Publisher Correction: Oviposition preference not necessarily predicts offspring performance in the fall armyworm, *Spodoptera frugiperda* (Lepidoptera: Noctuidae) on vegetable crops

**DOI:** 10.1038/s41598-021-97184-9

**Published:** 2021-08-26

**Authors:** Paola Sotelo-Cardona, Wen-Po Chuang, Mei-Ying Lin, Ming-Yao Chiang, Srinivasan Ramasamy

**Affiliations:** 1 World Vegetable Center, 60 Yi-Min Liao, Shanhua, Tainan, 74151 Taiwan, ROC; 2 National Taiwan University, No. 1, Sec. 4, Roosevelt Rd., Taipei, 10617 Taiwan, ROC; 3 Taiwan Agricultural Research Institute, Council of Agriculture, No.189, Zhongzheng Rd., Wufeng Dist., Taichung City, 413008 Taiwan, ROC

Correction to: *Scientific Reports* 10.1038/s41598-021-95399-4, published online 05 August 2021

Table 2 was truncated in the original version of the Article. This error has now been corrected in the PDF version of the Article; the HTML version was correct from the time of publication.

